# P2X7 Receptor Augments LPS-Induced Nitrosative Stress by Regulating Nrf2 and GSH Levels in the Mouse Hippocampus

**DOI:** 10.3390/antiox11040778

**Published:** 2022-04-13

**Authors:** Duk-Shin Lee, Ji-Eun Kim

**Affiliations:** Department of Anatomy and Neurobiology, Institute of Epilepsy Research, College of Medicine, Hallym University, Chuncheon 24252, Korea; dslee84@hallym.ac.kr

**Keywords:** ASCT2, glutamate-glutamine cycle, glutaminase, glutamine synthase, glutathione synthetase, iNOS, *S*-nitrosylated cysteine, xCT

## Abstract

P2X7 receptor (P2X7R) regulates inducible nitric oxide synthase (iNOS) expression/activity in response to various harmful insults. Since P2X7R deletion paradoxically decreases the basal glutathione (GSH) level in the mouse hippocampus, it is likely that P2X7R may increase the demand for GSH for the maintenance of the intracellular redox state or affect other antioxidant defense systems. Therefore, the present study was designed to elucidate whether P2X7R affects nuclear factor-erythroid 2-related factor 2 (Nrf2) activity/expression and GSH synthesis under nitrosative stress in response to lipopolysaccharide (LPS)-induced neuroinflammation. In the present study, P2X7R deletion attenuated iNOS upregulation and Nrf2 degradation induced by LPS. Compatible with iNOS induction, P2X7R deletion decreased *S*-nitrosylated (SNO)-cysteine production under physiological and post-LPS treated conditions. P2X7R deletion also ameliorated the decreases in GSH, glutathione synthetase, GS and ASCT2 levels concomitant with the reduced *S*-nitrosylations of GS and ASCT2 following LPS treatment. Furthermore, LPS upregulated cystine:glutamate transporter (xCT) and glutaminase in *P2X7R^+/+^* mice, which were abrogated by P2X7R deletion. LPS did not affect GCLC level in both *P2X7R^+/+^* and *P2X7R^−/−^* mice. Therefore, our findings indicate that P2X7R may augment LPS-induced neuroinflammation by leading to Nrf2 degradation, aberrant glutamate-glutamine cycle and impaired cystine/cysteine uptake, which would inhibit GSH biosynthesis. Therefore, we suggest that the targeting of P2X7R, which would exert nitrosative stress with iNOS in a positive feedback manner, may be one of the important therapeutic strategies of nitrosative stress under pathophysiological conditions.

## 1. Introduction

P2X7 receptor (P2X7R) is one of the cation-permeable ATP ligand-gated ion channels. P2X7R activation is involved in neuronal excitability, neuroinflammation and functions of astrocytes, as well as microglia [1,2,3,4]. P2X7R also regulates the generation of reactive oxygens species (ROS) and nitric oxide (NO) in response to various harmful insults [5,6]. Indeed, P2X7R activation enhances lipopolysaccharide (LPS)-induced inducible NO synthase (iNOS) expression [7,8,9]. NO can alter protein structure and function and exert biological effects directly by binding to free thiol groups on cysteine residues of target proteins forming *S*-nitrosylated (SNO)-proteins (known as *S*-nitrosylation) [10]. Furthermore, we have recently reported that *S*-nitrosylation of P2X7R facilitates its trafficking on the cell membrane [11]. Therefore, it is plausible that P2X7R may augment nitrosative stress with iNOS in a positive feedback manner, which would play an important role in the pathogenesis of various neurological diseases related to inflammations.

Glutathione (GSH) is an endogenous tripeptide (glutamate-cysteine-glycine) antioxidant. GSH is synthesized via several steps. Glutamate cysteine ligase (GCLC) is the rate-limiting enzyme that converts glutamate and cysteine (mostly derived from cystine; the oxidized dimer form of cysteine) to γ-glutamylcysteine. GSH synthetase (GSHS) adds glycine (derived from exogenous glycine or serine) to γ-glutamylcysteine for generating GSH in an ATP-driven reaction. Glutaminase (GLS) and glutamine synthase (GS) are also involved in GSH synthesis by regulating the glutamate-glutamine cycle [12]. In addition, some membrane transporters mediate GSH synthesis to supply neutral amino acids. Solute carrier 1 (SLC1) A4 and A5 known as ASCT1 and ASCT2, respectively, preferentially transfer the substrates **a**lanine, **s**erine and **c**ysteine (term ASC) [13]. Cystine:glutamate transporter (xCT or SLC7a11) exchanges cystine for glutamate (or cysteine) with a molar ratio of 1:1 by the substrate gradients across the plasma membrane under physiological conditions [14,15]. Interestingly, P2X7R activation decreases glutamate uptake and GS activity in astrocytes, although P2X7R cannot affect the release of GSH. Furthermore, P2X7R activation regulates xCT-mediated glutamate and ASCT2-mediated D-serine releases from astrocytes [4,14,15,16,17]. Therefore, it is plausible that P2X7R activity would negatively regulate GSH levels in the brain. However, we have recently reported that P2X7R deletion paradoxically decreases the basal GSH level in the mouse hippocampus, although it does not influence GCLC, GSHS and GLS expression levels [18]. Considering P2X7R-mediated generations of ROS and NO [5,6], it is likely that P2X7R deletion may reduce the demand of GSH for maintenance of the intracellular redox state or affect other antioxidant defense systems, which has been elusive.

Nuclear factor-erythroid 2-related factor 2 (Nrf2), a redox-sensitive transcription factor, regulates antioxidant-response element (ARE)-dependent transcription and the expression of antioxidant enzymes, which contributes to a broad spectrum of cellular functions, such as redox balance, cell cycle, cell death, immunity, metabolism, selective protein degradation, development, aging and carcinogenesis [19,20]. Under physiological conditions, a cytoplasmic repressor Kelch-like erythroid cell-derived protein with CNC homology (ECH)-associated protein 1 (Keap1) binds to Nrf2, which prevents nuclear Nrf2 translocation and mediates Nrf2 polyubiquitinylation and subsequent proteasomal degradation through the cullin-3 (Cul3)-based E3 ubiquitin ligase complex [21,22,23]. Oxidation and *S*-nitrosylation of SH-groups in Keap1 lead to the liberation of Nrf2 from Keap1 binding and increase Nrf2-mediated transactivation of multiple ARE-bearing genes, such as GSH synthetic enzymes [21,22,23]. Since LPS administration decreases GSH levels in the brain [24], it is presumable that P2X7R-mediated nitrosative stress may affect Nrf2 activity, which regulates GSH synthesis. Indeed, P2X7R deletion prevents the diminished efficacy of N-acetylcysteine (NAC, a GSH precursor) in GSH synthesis following SIN-1 (500 μM, a generator of nitric oxide, superoxide and peroxynitrite) treatment [18]. Furthermore, glutamine regulates both GSHS and Nrf2 levels [25]. However, it is unknown whether P2X7R-mediated nitrosative stress affects Nrf2 activity/expression and GSH synthesis in response to LPS-induced neuroinflammation.

Here, we demonstrate, for the first time, that P2X7R augmented nitrosative stress by Nrf2 degradation, impaired GSH synthesis, aberrant glutamate-glutamine cycle and dysfunctions of cysteine transporter following LPS treatment. Therefore, we suggest that P2X7R may be responsible for nitrosative stress in inflammatory conditions.

## 2. Materials and Methods

### 2.1. Experimental Animals, Chemicals and LPS Treatment

We used male C57BL/6J (*P2X7R^+/+^,* WT) and *P2X7R^−^^/^^−^* (KO) mice (60- to 90-day-old, 25–30 g, The Jackson Laboratory, USA) in the present study. Animals were given a commercial diet and water *ad libitum* under controlled conditions (22 °C ± 2 °C, 55% ± 5% humidity, and 12-h light/12-h dark cycle). All experimental protocols described below were approved by the Institutional Animal Care and Use Committee of Hallym University (Chuncheon, South Korea, Code number: Hallym 2018-3, approval date: 30 April 2018 and Hallym 2021-30, approval date: 17 May 2021). Every effort was made to reduce the number of animals employed and to minimize animal discomfort. All reagents were obtained from Sigma-Aldrich (St. Louis, MO, USA), except as noted. Animals were treated with LPS (5 mg/kg i.p.). Control animals received an equal volume of normal saline instead of LPS. Three days after LPS injection, animals were used for GSH assay, immunohistochemistry, Western blot and measurements of *S*-nitrosylation.

### 2.2. GSH Assay

Animals were sacrificed by decapitation. Hippocampal tissues were rapidly removed and sonicated with 0.5 mL of 5% sulfosalicylic acid and centrifuged at 10,000× *g* for 10 min at 4 °C. The supernatant was mixed with 1 mL of dithiobis-2-nitrobenzoic acid and 1 mL EDTA in 100 mL sodium phosphate buffer, pH 7.5, and 1 mL NADPH and 200 U/mL of glutathione reductase was added. GSH standards were treated identically, and optical absorbance of samples and standards was measured at 405 nm. Values were normalized to protein content as determined with a BCA protein assay kit (Thermo Scientific, Waltham, MA, USA) [18].

### 2.3. Immunohistochemistry

Animals were anesthetized with urethane anesthesia (1.5 g/kg, i.p.) and perfused transcardially with 4% paraformaldehyde in 0.1 M phosphate buffer (PB, pH 7.4). Brains were post-fixed in the same fixative overnight. Brain tissues were cryoprotected by infiltration with 30% sucrose overnight. Thereafter, Brains were cryosectioned at 30 μm. Free-floating sections were washed 3 times in PBS (0.1 M, pH 7.3) and incubated with 3% bovine serum albumin in PBS for 30 min at room temperature. Later, sections were incubated with a cocktail solution containing primary antibodies (Table 1) in PBS containing 0.3% Triton X-100 overnight at room temperature. Thereafter, sections were visualized with appropriate Cy2- and Cy3-conjugated secondary antibodies. Some tissues were incubated in biotinylated IgG and avidin-peroxidate complex and developed in 3,3′-diaminobenzidine in 0.1 M Tris buffer. Immunoreaction was observed using an Axio Scope microscope (Carl Zeiss Korea, Seoul, South Korea). To establish the specificity of the immunostaining, a negative control test was carried out with preimmune serum instead of the primary antibody. All experimental procedures in this study were performed under the same conditions and in parallel. To measure fluorescent intensity, five areas/animals (300 μm^2^/area) were randomly selected within the hippocampus (5 sections from each animal, *n* = 7 in each group). Thereafter, the mean intensity of each section was measured by using AxioVision Rel. 4.8 and ImageJ software. Intensity measurements were represented as the number of a 256 grayscale. The intensity of each section was standardized by setting the threshold level (mean background intensity obtained from five image inputs). Manipulation of the images was restricted to threshold and brightness adjustments to the whole image. 

### 2.4. Western Blot

Animals were decapitated under urethane anesthesia (1.5 g/kg, i.p.). The hippocampus was rapidly dissected out and homogenized in lysis buffer. The protein concentration in the supernatant was determined using a Micro BCA Protein Assay Kit (Pierce Chemical, Dallas, TX, USA). Thereafter, Western blot was performed by the standard protocol (*n* = 7 in each group). The primary antibodies used in the present study are listed in Table 1. The bands were detected and quantified on an ImageQuant LAS4000 system (GE Healthcare Korea, Seoul, South Korea). As an internal reference, rabbit anti-β-actin primary antibody (1:5000) was used. The values of each sample were normalized with the corresponding amount of β-actin. 

### 2.5. Measurement of S-Nitrosylation on GS and ASCT2 

Modified biotin switch assay was performed with the *S*-nitrosylation Western Blot Kit (ThermoFisher) according to the manufacturer’s protocol. Briefly, lysates were reacted with ascorbate in HENS buffer for specific labeling with iodoTMTzero reagents with MMT pretreatment. Protein labeling can be confirmed by Western blot using TMT antibodies. Thereafter, TMT-labeled proteins were purified by Anti-TMT Resin, eluted by TMT elution buffer, and identified by Western blot according to standard procedures. For technical controls, we omitted ascorbate for each sample. The ratio of SNO-protein to total protein was described as *S*-nitrosylation levels [11].

### 2.6. Data Analysis

Quantitative data are expressed as mean ± standard error of the mean. After the Shapiro–Wilk *W*-test was used to evaluate the values on normality, data were analyzed by the Student *t*-test, paired Student *t*-test, or one-way analysis of variance (ANOVA) followed by Newman–Keuls posthoc test. A *p* < 0.05 is considered to be statistically different.

## 3. Results

### 3.1. P2X7R Deletion Ameliorates Microglial Activation, but Not Reactive Astrogliosis in Response to LPS

**Figure 1:**
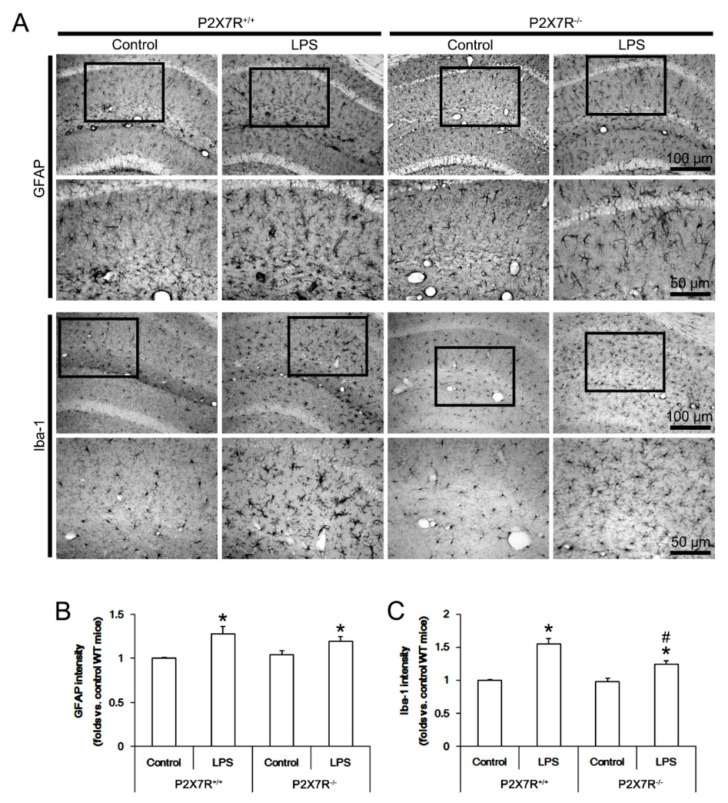
Effects of P2X7R deletion on microglial and astroglial responses to LPS. P2X7R deletion attenuates microglial activation, but not reactive astrogliosis induced by LPS. (**A**) Representative images for GFAP (an astroglial marker) and Iba-1 (a microglial marker) positive cells. Low panels are high magnification photos of boxes in upper panels. (**B**,**C**) Quantification of effects of P2X7R on GFAP and Iba-1 intensities following LPS treatment. Error bars indicate S.E.M. (*,^#^ *p* < 0.05 vs. control and WT mice, *n* = 7, respectively).

First, we evaluated the role of P2X7R in glial responses to LPS in the mouse hippocampus in vivo. In *P2X7R^+/+^* mice, LPS increased glial fibrillary acidic protein (GFAP, an astroglial marker) (*F*_(1,12)_ = 36.75, *p* < 0.001, one-way ANOVA, *n* = 7, respectively) and ionized calcium-binding adapter molecule-1 (Iba-1, a microglial marker) (*F*_(1,12)_ = 60.0, *p* < 0.001, one-way ANOVA, *n* = 7, respectively) intensities indicating reactive astrogliosis and microgliosis, respectively (Figure 1A–C). P2X7R deletion did not affect GFAP and Iba-1 intensities under physiological condition (Figure 1A–C) but attenuated the increased Iba-1 (*F*_(1,12)_ = 44.17, *p* < 0.001, one-way ANOVA, *n* = 7, respectively), but not GFAP, intensity induced by LPS (Figure 1A–C). These findings indicate that P2X7R may play an important role in microglial activation rather than reactive astrogliosis following LPS treatment.

### 3.2. P2X7R Deletion Attenuates LPS-Induced iNOS Induction in Microglia Rather Than Astrocytes

**Figure 2:**
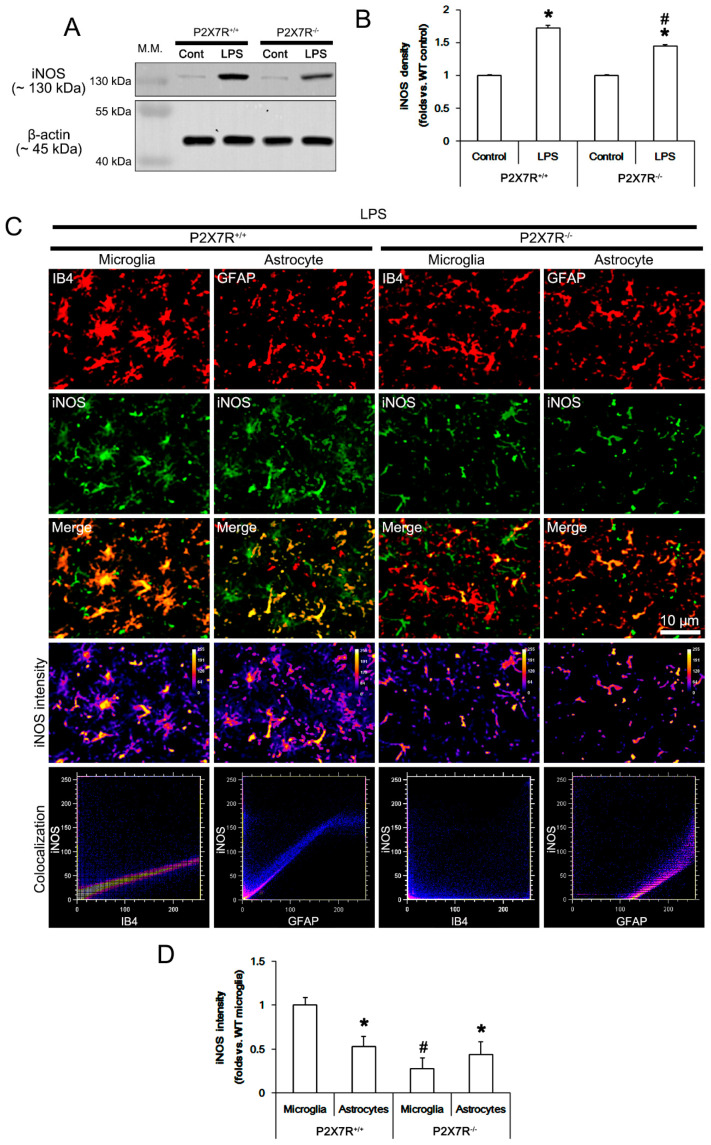
Effects of P2X7R deletion on LPS-induced iNOS induction in microglia and astrocytes. P2X7R deletion ameliorates iNOS induction in microglia rather than astrocytes following LPS injection. (**A**) Representative Western blot of iNOS in the whole hippocampus. (**B**) Quantification of iNOS protein level based on Western blot data. Error bars indicate S.E.M. (*,^#^ *p* < 0.05 vs. control and WT mice, *n* = 7, respectively). (**C**) Representative photos of iNOS expression, intensity and the degree of colocalization in IB4 (a microglial marker) and GFAP (an astroglial marker) positive cells. (**D**) Quantification of iNOS induction in microglia and astrocytes. Error bars indicate S.E.M. (*,^#^ *p* < 0.05 vs. control and WT mice, *n* = 7, respectively). Full-length gel images of Western blot data in (**A**) could be found in Supplementary Figure S1.

P2X7R activation increases NO production in response to LPS [7], while oxidized ATP (OxATP, a P2X7R antagonist) blocks LPS-induced NO production in vitro [26]. Therefore, we investigated the effect of P2X7R deletion on iNOS induction in responses to LPS in vivo. Under physiological conditions, no difference in iNOS protein levels between *P2X7R^+/+^* and *P2X7R^−/−^* mice (Figure 2A,B). LPS increased iNOS expression to 1.72- and 1.45-fold of control level in *P2X7R^+/+^* and *P2X7R^−/−^* mice, respectively (*F*_(3,24)_ = 163.7, *p* < 0.001, one-way ANOVA, *n* = 7, respectively; Figure 2A,B). LPS-induced iNOS induction was lower in *P2X7R^−/−^* mice than that in *P2X7R^+/+^* mice (*F*_(1,12)_ = 27.3, *p* < 0.001, one-way ANOVA, *n* = 7, respectively; Figure 2A,B). An immunohistochemical study revealed that LPS led to iNOS induction in microglia and astrocytes in *P2X7R^+/+^* mice (Figure 2C). LPS-induced iNOS upregulation was higher in microglia than that in astrocytes (*t*_(6)_ = 12.7, *p* < 0.001, paired Student *t*-test, *n* = 7, respectively; Figure 2C,D). In *P2X7R^−/−^* mice, LPS-induced iNOS expression in microglia was 0.28-fold of *P2X7R^+/+^* mice level (*F*_(1,12)_ = 170.5, *p* < 0.001, one-way ANOVA, *n* = 7, respectively; Figure 2C,D). Astroglial iNOS level in *P2X7R^−/−^* mice was similar to that in *P2X7R^+/+^* mice. Thus, iNOS level was higher in astrocytes than that in microglia unlike *P2X7R^+/+^* mice (*t*_(6)_ = 3.27, *p* = 0.02, paired Student *t*-test, *n* = 7, respectively; Figure 2C,D). These findings indicate that P2X7R may enhance iNOS induction in microglia more than astrocytes following LPS treatment. 

### 3.3. P2X7R Deletion Attenuates LPS-Induced SNO-Cysteine Production in Microglia and Astrocytes

**Figure 3:**
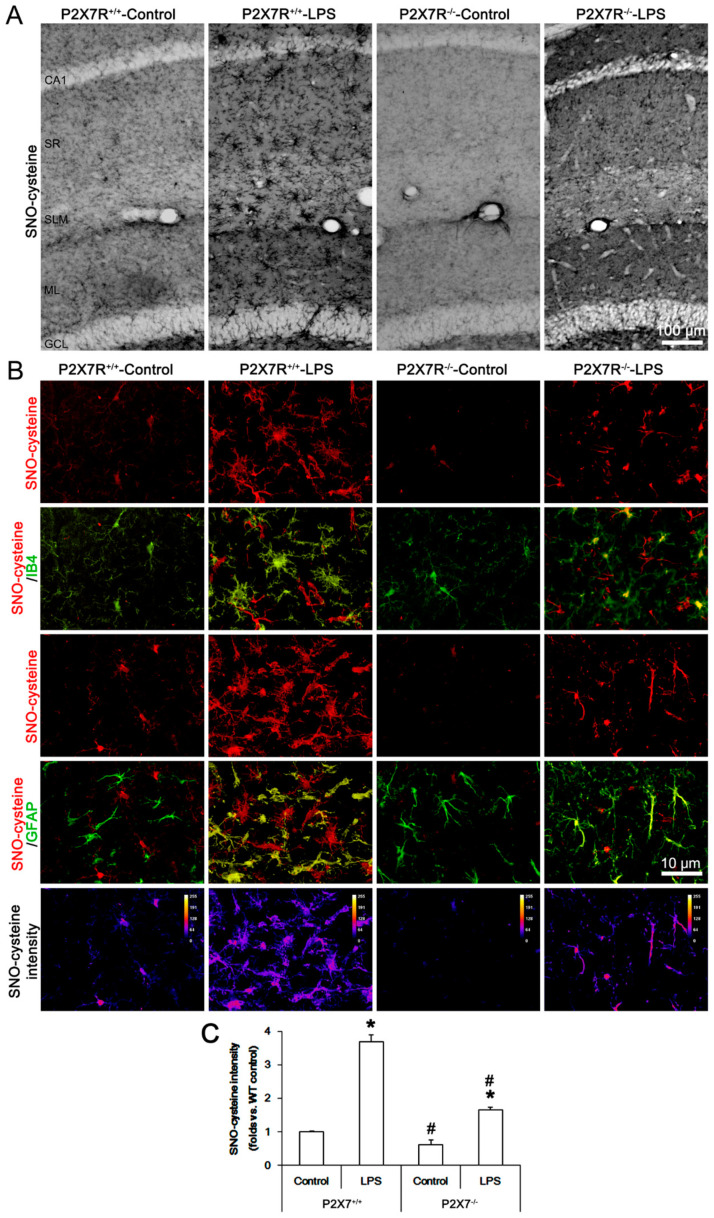
Effects of P2X7R deletion on LPS-induced SNO-cysteine production in microglia and astrocytes. Under physiological conditions, SNO-cysteine level in the hippocampus is higher in *P2X7R^+/+^* mice than that in *P2X7R^−/−^* mice. LPS increases SNO-cysteine production in microglia and astrocytes within the hippocampus of *P2X7R^+/+^* more than *P2X7R^−/−^* mice. In *P2X7R^−/−^* mice, SNO-cysteine level is lower in microglia than that in astrocytes. (**A**) Representative images for SNO-cysteine in the hippocampus. (**B**) Representative photos of SO-cysteine production in IB4 (a microglial marker) and GFAP (an astroglial marker) positive cells. (**C**) Quantification of SNO-cysteine production in the hippocampus. Error bars indicate S.E.M. (*,^#^ *p* < 0.05 vs. control and WT mice, *n* = 7, respectively).

To confirm the effects of P2X7R deletion on iNOS-mediated NO synthesis, we performed the immunohistochemical study using an antibody detecting SNO-cysteine in vivo. Under physiological conditions, SNO-cysteine level in the hippocampus was higher in *P2X7R^+/+^* mice than that in *P2X7R^−/−^* mice (Figure 3A). Double immunofluorescent data demonstrated that SNO-cysteine signal was mainly detected in microglia in *P2X7R^+/+^* mice, while it was weakly observed in *P2X7R^−/−^*mice (Figure 3B). Following LPS treatment, SNO-cysteine levels were increased in microglia and astrocytes in *P2X7R^+/+^* and *P2X7R^−/−^* mice (Figure 3B,C). LPS increased SNO-cysteine production to 3.7- and 1.65-fold of control level in *P2X7R^+/+^* and *P2X7R^−/−^* mice, respectively (*F*_(3,24)_ = 109.1, *p* < 0.001, one-way ANOVA, *n* = 7, respectively; Figure 3B,C), indicating that LPS-induced SNO-cysteine production was significantly lower in *P2X7R^−/−^* mice than that in *P2X7R^+/+^* mice (*F*_(1,12)_ = 65.6, *p* < 0.001, one-way ANOVA, *n* = 7, respectively; Figure 3B,C). These findings indicate that P2X7R may reinforce LPS-induced iNOS upregulation that would increase SNO-cysteine production. 

### 3.4. P2X7R Deletion Ameliorates LPS-Induced Nrf2 Downregulation

**Figure 4:**
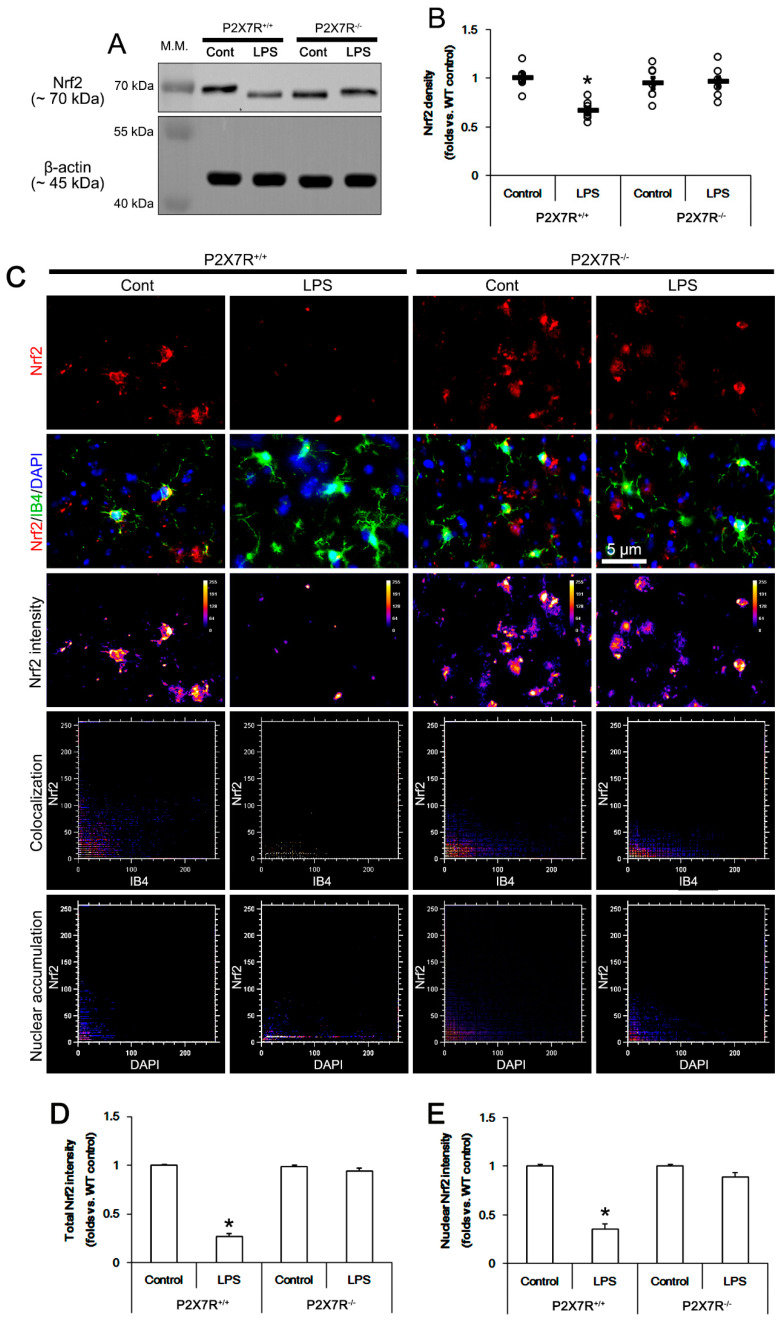
Effects of P2X7R deletion on LPS-induced Nrf2 downregulation in microglia. LPS decreases Nrf2 protein level in the hippocampus of *P2X7R^+/+^* mice, but not *P2X7R^−/−^* mice, since total Nrf2 level and its nuclear accumulation are reduced in microglia. (**A**) Representative Western blot of Nrf2 in the whole hippocampus. (**B**) Quantification of iNOS protein level based on Western blot data. Open circles indicate each individual value. Horizontal and error bars indicate the mean value and S.E.M., respectively (*,^#^ *p* < 0.05 vs. control and WT mice, *n* = 7, respectively). (**C**) Representative photos of Nrf2 expression, intensity and the degree of colocalization in IB4 (a microglial marker) positive cells and DAPI (a nuclear marker). (**D**,**E**) Quantification of total and nuclear Nrf2 intensity in microglial. Error bars indicate S.E.M. (* *p* < 0.05 vs. control and WT mice, *n* = 7, respectively). Full-length gel images of Western blot data in this figure could be found in Supplementary Figure S2.

**Figure 5:**
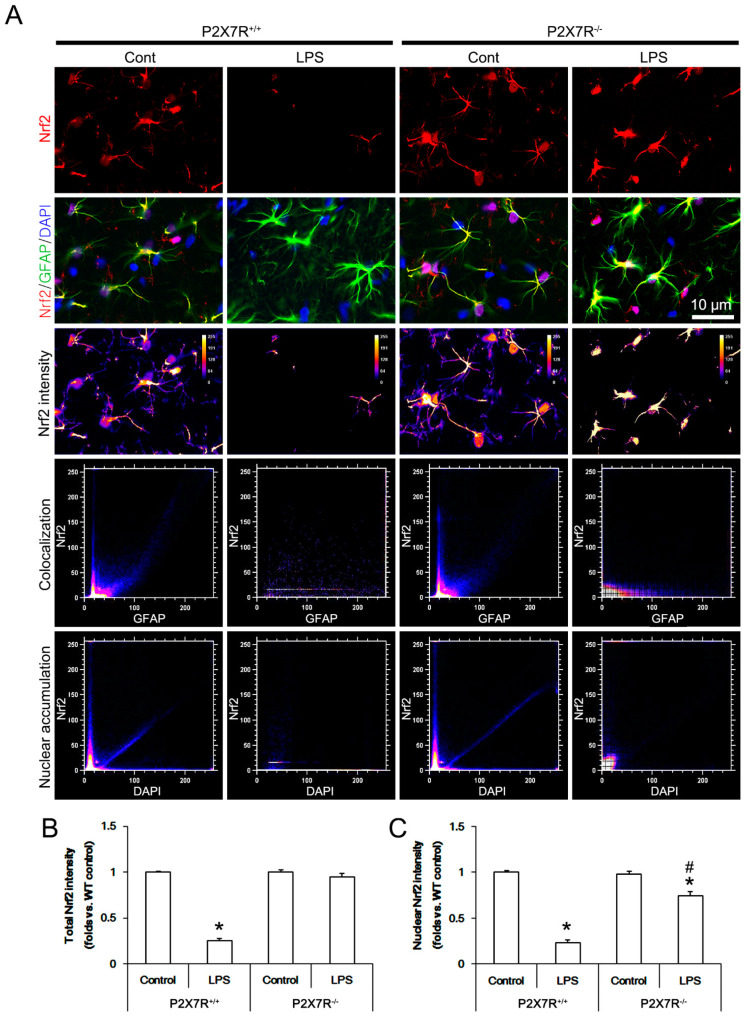
Effects of P2X7R deletion on LPS-induced Nrf2 downregulation in astrocytes. LPS decreases total Nrf2 protein level in astrocytes of *P2X7R^+/+^* mice, while it does not in astrocytes of *P2X7R^−/−^* mice. LPS also diminishes nuclear Nrf2 protein level in astrocytes of *P2X7R^+/+^* mice. LPS-induced Nrf2 downregulation in astrocytes is attenuated in *P2X7R^−/−^* mice. (**A**) Representative photos of Nrf2 expression, intensity and the degree of colocalization in GFAP (an astroglial marker) positive cells and DAPI (a nuclear marker). (**B**,**C**) Quantification of total and nuclear Nrf2 intensity in astrocytes. Error bars indicate S.E.M. (*,^#^ *p* < 0.05 vs. control and WT mice, *n* = 7, respectively).

LPS elevates ROS level [27,28], which subsequently decreases Nrf2 level [29]. Furthermore, Nrf2 activation (nuclear accumulation) effectively inhibits LPS-induced iNOS upregulation [30,31]. Therefore, it is likely that P2X7R may facilitate LPS-induced iNOS expression via Nrf2 downregulation, which has been unknown. Thus, we validated Nrf2 protein level in the hippocampi of *P2X7R^+/+^* and *P2X7R^−/−^* mice following LPS treatment. Under physiological condition, there was no difference in Nrf2 protein level between *P2X7R^+/+^* and *P2X7R^−/−^* mice (*F*_(1,12)_ = 0.463, *p* = 0.51, one-way ANOVA, *n* = 7, respectively; Figure 4A,B). LPS decreased total Nrf2 protein level to 0.74-fold of control level in *P2X7R^+/+^* mice (*F*_(1,12)_ = 33.177, *p* < 0.001, one-way ANOVA, *n* = 7, respectively; Figure 4A,B), but not *P2X7R^−/−^* mice (*F*_(1,12)_ = 0.025, *p* = 0.878, one-way ANOVA, *n* = 7, respectively; Figure 4A,B). LPS decreased total Nrf2 level and its nuclear accumulation in microglia to 0.27- and 0.35-fold of control level in *P2X7R^+/+^* mice, respectively, (*F*_(1,12)_ = 318.7 and 84.2, *p* < 0.001, respectively, one-way ANOVA, *n* = 7, respectively; Figure 4C–E) but not in *P2X7R^−/−^* mice (Figure 4C–E). LPS also reduced total Nrf2 level and its nuclear accumulation in astrocytes to 0.25- and 0.23-fold of control level in *P2X7R^+/+^* mice, respectively (*F*_(1,12)_ = 461.8 and 428.6, *p* < 0.001, respectively, one-way ANOVA, *n* = 7, respectively; Figure 5A–C). In *P2X7R^−/−^* mice, LPS declined nuclear Nrf2 accumulation in astrocytes to 0.74-fold of control level without altering total Nrf2 level (*F*_(1,12)_ = 15.2, *p* = 0.002, one-way ANOVA, *n* = 7, respectively; Figure 5A–C). These findings indicate that P2X7R may facilitate the decreases in total Nrf2 level and its nuclear accumulation in microglia and astrocytes following LPS treatment. 

### 3.5. P2X7R Aggravates the Decreased GSH Concentration Induced by LPS 

**Figure 6:**
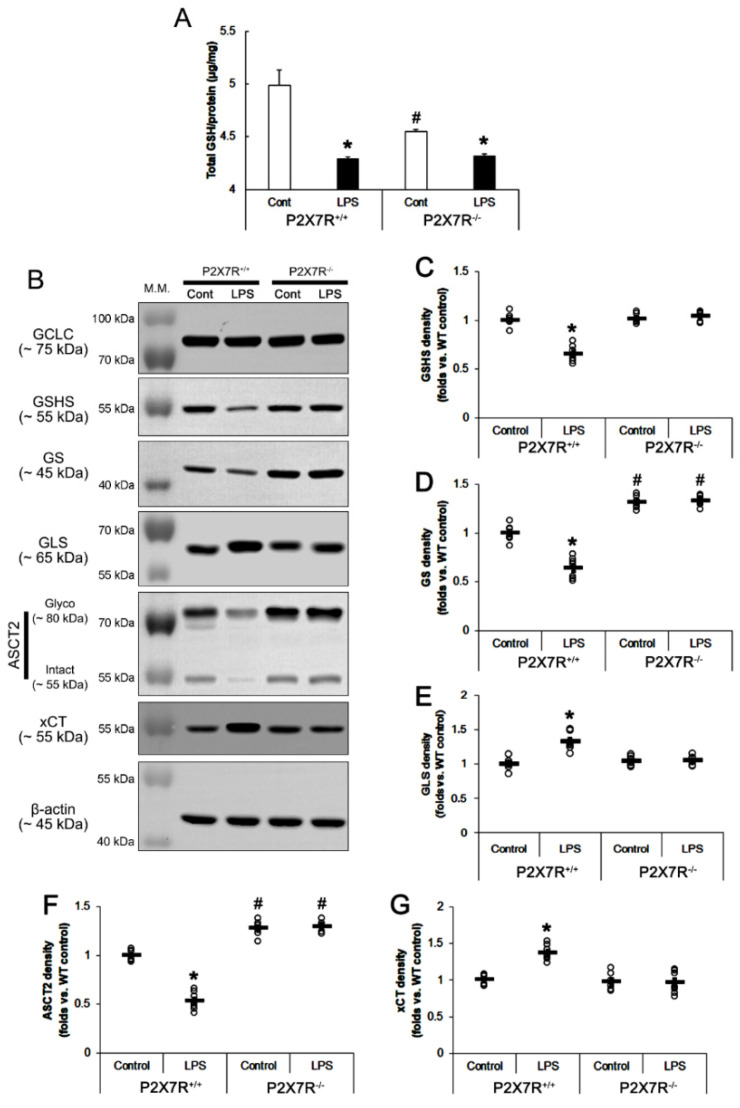
Effects of P2X7R deletion on GSH concentration and expressions of GCLC, GSHS, GS, GLS, ASCT2 and xCT following LPS injection. Under physiological condition, P2X7R deletion reduces GSH level in the hippocampus. However, P2X7R deletion increases GS and ASCT2 levels. LPS declines GSH concentration in *P2X7R^+/+^* mice more than in *P2X7R^−/−^* mice. LPS decreases GSHS, GS and ASCT2 levels, but increases GLS and xCT levels only in the *P2X7R^+/+^* mice. (**A**) Total GSH level in the hippocampus under physiological and post-LPS treated conditions. (**B**) Representative Western blot of GCLC, GSHS, GS, GLS, ASCT2 and xCT in the whole hippocampi of that *P2X7R^+/+^* and *P2X7R^−/−^* mice. (**C**–**G**) Quantification of GSHS, GS, GLS, ASCT2 and xCT levels based on Western blot data. Open circles indicate each individual value. Horizontal and error bars indicate the mean value and S.E.M., respectively (*,^#^ *p* < 0.05 vs. control and WT mice, *n* = 7, respectively). Full-length gel images of Western blot data in (**B**) could be found in Supplementary Figure S3.

Recently, we have reported that P2X7R deletion reduces the total GSH level in the hippocampus by regulating the glutamate-glutamine cycle and neutral amino acid transports under physiological conditions, which may be a consequent response to the absence of P2X7R-mediated oxidative or nitrosative stresses [18]. Since Nrf2 plays a key role in the regulation of GSH synthesis [32], it is likely that P2X7R-mediated Nrf2 downregulation may influence GSH levels following LPS injection. Consistent with our previous study [18], the present data showed that total GSH level in *P2X7R^−/−^* mice (4.55 ± 0.02 μg/mg protein) was lower than that in *P2X7R^+/+^* mice (5 ± 0.14 μg/mg protein; *F*_(1,12)_ = 21.361, *p* < 0.001; one-way ANOVA, *n* = 7, respectively; Figure 6A). LPS decreased total GSH concentration in *P2X7R^+/+^* mice (4.29 ± 0.02 μg/mg protein, 86% of control level; *F*_(1,12)_ = 11.618, *p* = 0.005, *n* = 7, respectively) more than *P2X7R^−/−^* mice (4.31 ± 0.02 μg/mg protein, 95% of control level; *F*_(1,12)_ = 19.835, *p* < 0.001, *n* = 7, respectively). Thus, there was no difference in total GSH level in both groups following LPS treatment (Figure 6A). Considering GSH decreases LPS-induced NO production by inhibiting iNOS expression [33], our findings indicate that LPS-induced nitrosative stress may be more severe in *P2X7R^+/+^* mice and lead to the higher GSH consumption than those in *P2X7R^−/−^* mice. 

### 3.6. P2X7R Downregulates GSHS, but Not GCLC Expression following LPS Treatment

Next, we explored if P2X7R deletion would also influence GSH production. GCLC is the rate-limiting enzyme in GSH biosynthesis [34]. Thus, we investigated whether P2X7R deletion and/or LPS affect GCLC expression in the mouse hippocampus. Under physiological conditions, GCLC expression level was similarly observed in *P2X7R^+/+^* and *P2X7R^−/−^* mice. Consistent with previous studies [35,36], LPS did not affect GCLC expression levels in both *P2X7R^+/+^* and *P2X7R^−/−^* mice (*F*_(3,24)_ = 0.12, *p* = 0.95, one-way ANOVA, *n* = 7, respectively; Figure 6B).

GSHS catalyzes *γ*-glutamylcysteine and glycine to GSH. The present data showed that P2X7R deletion did not affect GSHS expression under physiological conditions. Consistent with a previous study demonstrating a marked reduction in GSHS expression induced by LPS [37], *P2X7R^+/+^* mice showed the decreased GSHS expression (66% of control level) in the hippocampus following LPS treatment, while *P2X7R^−/−^* mice did not (*F*_(3,24)_ = 55.67, *p* < 0.001, one-way ANOVA, *n* = 7, respectively; Figure 6B,C). These findings indicate that P2X7R deletion may attenuate the decreased GSH concentration by maintaining GSHS expression following LPS treatment. 

### 3.7. P2X7R Downregulates GS, but Increases GLS Expression following LPS Treatment

Glutamate and glutamine are equally used as precursors for GSH synthesis in astrocytes, which is regulated by Nrf2 [32]. GS catalyzes the conversion of glutamate and ammonia to glutamine and plays a major role in ammonia detoxification, interorgan nitrogen flux, acid–base regulation, cell proliferation and protection from apoptotic stimuli [38]. Recently, we have reported that GS expression is higher in *P2X7R^−/−^* mice than *P2X7R^+/+^* mice under physiological conditions [18]. Thus, we investigated whether LPS distinctly influences GS expression between *P2X7R^+/+^* and *P2X7R^−/−^* mice. Consistent with our previous studies [18], GS expression in *P2X7R^−/−^* mice was 1.31-fold higher than that in *P2X7R^+/+^* mice under physiological condition (*t*_(12)_ = 7.871, *p* < 0.001, Student *t*-test, *n* = 7, respectively; Figure 6B,D). LPS decreased GS protein level to 0.66-fold of control level in *P2X7R^+/+^* mice, but not *P2X7R^−/−^* mice (*F*_(3,24)_ = 115.13, *p* < 0.001, one-way ANOVA, *n* = 7, respectively; Figure 6B,D). These findings indicate that LPS may reduce GS protein levels, which may be abrogated by P2X7R deletion. 

Astrocytes use glutamine as a precursor for GSH synthesis via the glutamate-glutamine cycle mediated by GLS and GS [39]. Since LPS activates GLS activity/expression [40], we also validated the effect of LPS on GLS protein levels in *P2X7R^+/+^* and *P2X7R^−/−^* mice. Under physiological conditions, there was no difference in GLS expression between *P2X7R^+/+^* and *P2X7R^−/−^* mice (Figure 6B,E). LPS increased GLS protein level in *P2X7R^+/+^*, but not *P2X7R^−/−^* mice (*F*_(3,24)_ = 19.16, *p* < 0.001, one-way ANOVA, *n* = 7, respectively; Figure 6B,E). Together with the altered GS expression, our findings suggest that P2X7R deletion may inhibit the changed glutamate-glutamine cycle induced by LPS.

### 3.8. P2X7R Upregulates xCT, but Decreases ASCT2 Expression following LPS Treatment

The increased GS expression and glutamine concentration potentially facilitate glutamine efflux from astrocytes by inducing the trafficking of ASCT2 [41,42]. Since P2X7R deletion increases ASCT2 expression [18], we investigated whether P2X7R affects ASCT2 expression following LPS treatment. In the present study, ASCT2 expression in *P2X7R^−/−^* mice was 1.33-fold higher than that in *P2X7R^+/+^* mice under physiological condition (*t*_(12)_ = 7.71, *p* < 0.001, Student *t*-test, *n* = 7, respectively; Figure 6B,F). LPS decreased ASCT2 protein expression to 0.53-fold of control level in *P2X7R^+/+^* mice, but not *P2X7R^−/−^* mice (*F*_(3,24)_ = 171.12, *p* < 0.001, one-way ANOVA, *n* = 7, respectively; Figure 6B,F). These findings indicate that P2X7R may inhibit ASCT2-mediated cysteine uptake and glutamine efflux.

xCT also influences GSH synthesis to supply neutral amino acids [14,15]. Thus, we explored whether LPS affects xCT protein levels in *P2X7R^+/+^* and *P2X7R^−/−^* mice. Under physiological conditions, xCT expression level was similarly detected in *P2X7R^+/+^* and *P2X7R^−/−^* mice (Figure 6B,G). However, LPS increased xCT expression levels in *P2X7R^+/+^* (137% of control level), but not *P2X7R^−/−^* mice (*F*_(3,24)_ = 20, *p* < 0.001, one-way ANOVA, *n* = 7, respectively; Figure 6B,G). Considering the changed ASCT2 expression, our findings indicate that P2X7R may inhibit GSH synthesis via dysfunction of cysteine uptake as well as aberrant glutamate-glutamine cycle following LPS treatment. 

### 3.9. P2X7R Regulates S-Nitrosylation of GS and ASCT2 under Physiological and Post-LPS Treated Condition

**Figure 7:**
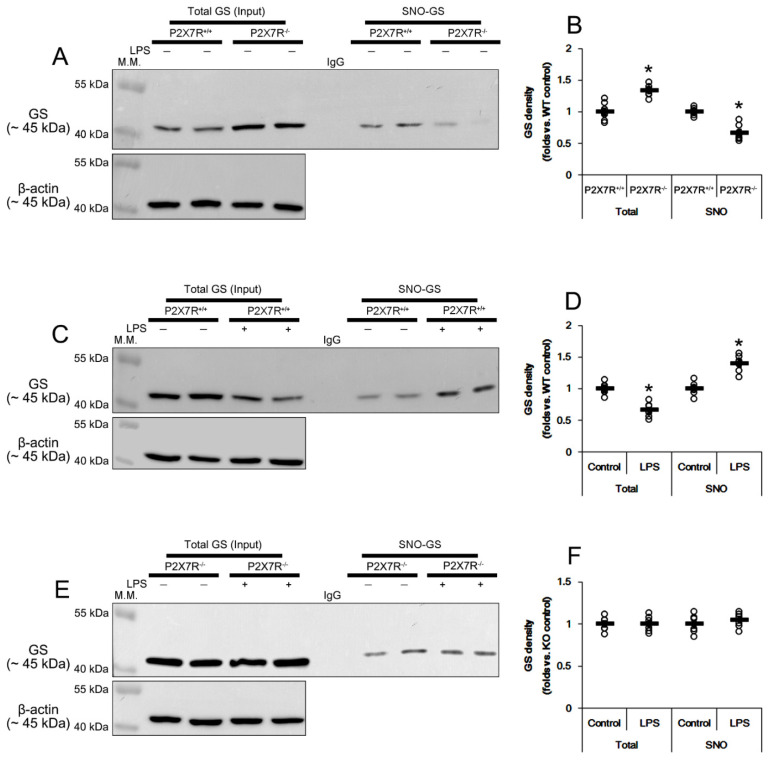
Effects of P2X7R deletion on S-nitrosylation of GS following LPS injection. Under physiological conditions, total GS level in *P2X7R^−/−^* mice is higher than that of *P2X7R^+/+^* mice. However, the SNO-GS level in *P2X7R^−/−^* mice is lower than that of *P2X7R^+/+^* mice. LPS decreases total GS level but increases SNO-GS level in *P2X7R^+/+^* mice. LPS does not affect them in *P2X7R^−/−^* mice. (**A**) Representative Western blot of total- and SNO-GS in the whole hippocampi of that *P2X7R^+/+^* and *P2X7R^−/−^* mice. (**B**) Quantification of the total- and SNO-GS level based on Western blot data. Open circles indicate each individual value. Horizontal and error bars indicate the mean value and S.E.M., respectively (* *p* < 0.05 vs. WT mice, *n* = 7, respectively). (**C**) Representative Western blot of total- and SNO-GS in the whole hippocampi of that *P2X7R^+/+^* mice following LPS treatment. (**D**) Quantification of total- and SNO-GS level based on Western blot data (* *p* < 0.05 vs. control mice, *n* = 7, respectively). (**E**) Representative Western blot of total- and SNO-GS in the whole hippocampi of that *P2X7R^−/−^* mice following LPS treatment. (**F**) Quantification of total- and SNO-GS level based on Western blot data. Horizontal and error bars indicate the mean value and S.E.M., respectively (*n* = 7, respectively). Full-length gel images of Western blot data in this figure could be found in Supplementary Figure S4.

**Figure 8:**
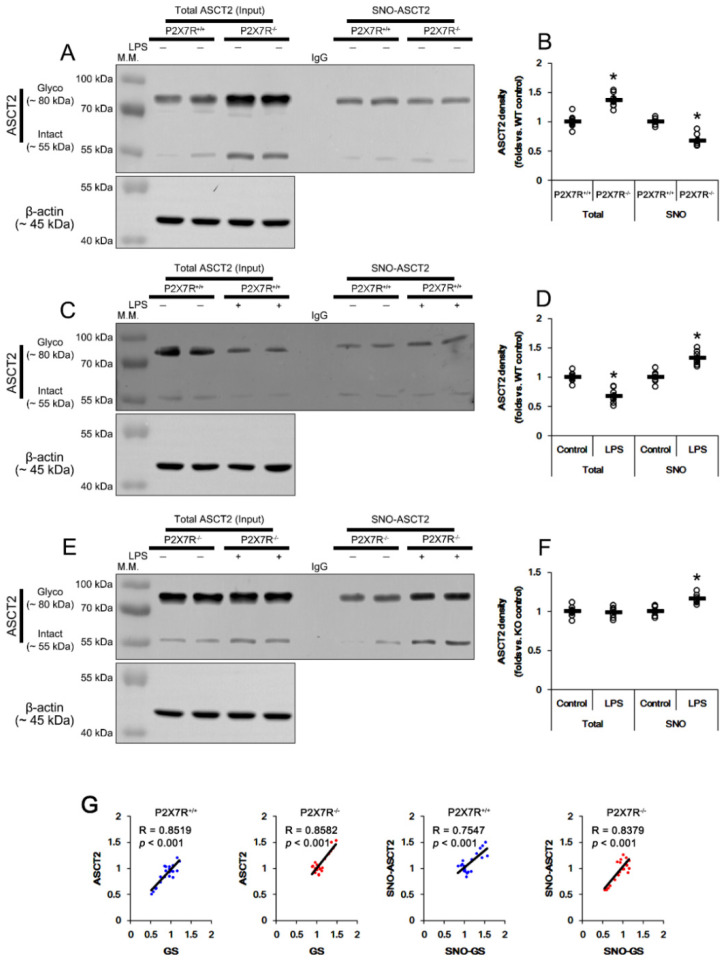
Effects of P2X7R deletion on ASCT2 expression and its S-nitrosylation following LPS injection. Under physiological conditions, total ASCT2 level in *P2X7R^−/−^* mice is higher than that of *P2X7R^+/+^* mice. However, SNO-ASCT2 level in *P2X7R^−/−^* mice is lower than that of *P2X7R^+/+^* mice. LPS decreases total ASCT2 level but increases SNO-ASCT2 level in *P2X7R^+/+^* mice. LPS also increases SNO-ASCT2 level in *P2X7R^−/−^* mice without affecting total ASCT2 level. Total- and SNO-ASCT2 levels show a direct proportional relationship with Total- and SNO-GS levels in *P2X7R^+/+^* and *P2X7R^−/−^* mice, respectively. (**A**) Representative Western blot of total- and SNO- ASCT2 in the whole hippocampi of that *P2X7R^+/+^* and *P2X7R^−/−^* mice. (**B**) Quantification of total- and SNO-ASCT2 level based on Western blot data. Open circles indicate each individual value. Horizontal and error bars indicate the mean value and S.E.M., respectively (* *p* < 0.05 vs. WT mice, *n* = 7, respectively). (**C**) Representative Western blot of total- and SNO-ASCT2 in the whole hippocampi of that *P2X7R^+/+^* mice following LPS treatment. (**D**) Quantification of total- and SNO-ASCT2 level based on Western blot data (* *p* < 0.05 vs. control mice, *n* = 7, respectively). (**E**) Representative Western blot of total- and SNO-ASCT2 in the whole hippocampi of that *P2X7R^−/−^* mice following LPS treatment. (**F**) Quantification of total- and SNO-ASCT2 level based on Western blot data (* *p* < 0.05 vs. control mice, *n* = 7, respectively). (**G**) Linear regression analyses of total- and SNO proteins between ASCT2 and GS in *P2X7R^+/+^* and *P2X7R^−/−^* mice. Full-length gel images of Western blot data in this figure could be found in Supplementary Figure S5.

NO can deplete GSH levels by *S*-nitrosylation of GSH metabolic enzymes [43]. Furthermore, four cysteine residues of GS are *S*-nitrosylated by NO: cysteine 99, 183, 269 and 346 [44]. Indeed, GS activity is highly susceptible to reactive nitrogen and oxygen species, and the inhibition of NO synthesis increases GS activity in rat brain and cultured rat astrocytes [45,46]. On the other hand, NO inhibits ASCT2 transporter activity by oxidation of cysteine residues [47]. To compensate NO-mediated ASCT2 inhibition, NO upregulates ASCT2 protein via *de novo* synthesis in vitro [48]. Thus, we also explored whether P2X7R deletion affects *S*-nitrosylation of GS and ASCT2 induced by LPS. Under physiological conditions, SNO-GS level in *P2X7R^−/−^* mice was 0.67-fold of *P2X7R^+/+^* mice level (*t*_(12)_ = 6.352, *p* < 0.001, Student *t*-test, *n* = 7, respectively; Figure 7A,B). LPS increased SNO-GS level to 1.4-fold of control level in *P2X7R^+/+^* mice (*t*_(12)_ = 6.48, *p* < 0.001, Student *t*-test, *n* = 7, respectively; Figure 7C,D), but not *P2X7R^−/−^* mice (*t*_(12)_ = 0.98, *p* = 0.346, Student *t*-test, *n* = 7, respectively; Figure 7E,F). These findings indicate that P2X7R deletion may ameliorate *S*-nitrosylation of GS under physiological- and post-LPS conditions. Similar to the *S*-nitrosylation of GS, SNO-ASCT2 level in *P2X7R^−/−^* mice was 0.68-fold of *P2X7R^+/+^* mice level under physiological condition (*t*_(12)_ = 6.475, *p* < 0.001, Student *t*-test, *n* = 7, respectively; Figure 8A,B). However, LPS increased SNO-ASCT2 level to 1.32- and 1.16-fold of control level in *P2X7R^+/+^* (*t*_(12)_ = 5.349, *p* < 0.001, Student *t*-test, *n* = 7, respectively; Figure 8C,D) and *P2X7R^−/−^* mice (*t*_(12)_ = 5.16, *p* < 0.001, Student *t*-test, *n* = 7, respectively; Figure 8E,F), respectively. Thus, P2X7R deletion attenuated increased SNO-ASCT2 production induced by LPS (*F*_(1,12)_ = 9.338, *p* = 0.01, one-way ANOVA, *n* = 7, respectively; Figure 8E,F).

Considering that the increased GS expression induces ASCT2 trafficking [41,42], we also analyzed the correlations of expression/*S*-nitrosylation level between GS and ASCT2. Linear regression analysis showed a direct proportional relationship between GS and ASCT2 levels with linear correlation coefficients of 0.8519 (*t*_(19)_ = 7.09, *p* < 0.001, *n* = 21, respectively; Figure 8G) and 0.8582 (*t*_(19)_ = 7.28, *p* < 0.001, *n* = 21, respectively; Figure 8G) in *P2X7R^+/+^* and *P2X7R^−/−^* mice, respectively. The SNO-GS level also showed a direct proportional relationship with SNO-ASCT2 level in *P2X7R^+/+^* (linear correlation coefficients, 0.7547; *t*_(19)_ = 5.01, *p* < 0.001, *n* = 21, respectively; Figure 8G) and *P2X7R^−/−^* mice (linear correlation coefficients, 0.8379; *t*_(19)_ = 6.69, *p* < 0.001; Figure 8G), respectively. These findings indicate that P2X7R may be involved in GS-mediated ASCT2 regulation under physiological and post-LPS treated conditions.

## 4. Discussion

LPS is a gram-negative bacterial cell surface proteoglycan, which triggers neuroinflammation via toll-like receptor 4 (TLR4)-mediated microglial and astroglial activation in the brain [49]. After exposure to LPS, P2X7R augments iNOS expression and production of NO in microglia and astrocytes [26,50]. Therefore, P2X7R is one of the modulators of neuroinflammatory responses. In the present study, SNO-cysteine level in the hippocampus was higher in *P2X7R^+/+^* mice than that in *P2X7R^−/−^* mice under physiological conditions. Furthermore, LPS led to microglial activation and reactive astrogliosis in *P2X7R^+/+^* mice concomitant with increases in iNOS expression and SNO-cysteine production, which were attenuated by P2X7R deletion. These findings indicate that P2X7R may regulate nitrosative stress in the brain under physiological and inflammatory conditions. Interestingly, *S*-nitrosylation facilitates the trafficking of P2X7R, which promotes microglial activation and astroglial dysfunction following status epilepticus (a sustained seizure activity) [11]. Therefore, our findings suggest that P2X7R and iNOS may exert nitrosative stress in a positive feedback manner under inflammatory conditions.

Nrf2 plays a role in the regulation of cellular redox homeostasis [32]. Under nitrosative stress, Nrf2 sequestered by Keap1 is transported to the nucleus and promotes ARE-related gene expressions. However, excessive nitrosative stress leads to Nrf2 proteasomal degradation [51,52]. Indeed, a low dose of LPS (0.5 mg/kg) increases Nrf2 expression accompanied by the upregulated heme oxygenase-1 (HO-1, one of the downstream genes of Nrf2) at 4 h after treatment [53]. However, a high dose of LPS (1 mg/kg/day) leads to Nrf2 downregulation 6 days after administration [54]. Furthermore, a high dose of LPS (1 mg/kg) decreases Nrf2 expression and is coupled with reduced HO-1 expression and the upregulations of interleukin-6 that are regulated by Nrf2 [54]. In the present study, LPS (5 mg/kg) decreased the total Nrf2 protein level and its nuclear accumulation in microglia and astrocytes 3 days after treatment, which are ameliorated by P2X7R deletion. Considering these previous studies and the present data, it is plausible that Nrf2 upregulation may be an adaptive response against oxidative and/or nitrosative stress in response to a low dose of LPS at the early time window. In contrast, a high dose of LPS would lead to Nrf2 downregulation (or degradation), and in turn a decreased Nrf2 capacity for defending against oxidative- or nitrosative stress would contribute to LPS-induced neuroinflammation at the late time window. Since Nrf2 inhibits iNOS upregulation and attenuates the formation of SNO-proteins induced by LPS [10,30,31]; therefore, our findings suggest that P2X7R may reinforce iNOS-mediated nitrosative stress by facilitating Nrf2 degradation following LPS treatment.

Recently, we have reported that P2X7R deletion reduces the total GSH level in the hippocampus under physiological conditions, as an adaptive response to the absence of oxidative or nitrosative stresses mediated by P2X7R [18]. Consistent with this report, the present data show that the total GSH level in *P2X7R^−/−^* mice was lower than that in *P2X7R^+/+^* mice. Furthermore, LPS decreased total GSH concentration to 86% and 95% of control levels in *P2X7R^+/+^* and *P2X7R^−/−^* mice, respectively. Regarding that Nrf2 activates GSH biosynthesis [55,56] and P2X7R deletion prevented LPS-induced GSHS downregulation in the present study, our findings indicate that P2X7R may aggravate LPS-induced nitrosative stress, which would increase GSH consumption or reduce GSH synthesis. 

On the other hand, Nrf2 activates GCLC and xCT which maintain intracellular GSH levels by regulating the rate-limiting steps for GSH synthesis. Furthermore, GSH depletion increases the transcription of *Nrf2* and *xCT* [55,56,57]. Therefore, GSH concentration and the Nrf2 system may be reciprocally regulated by each other. In the present study, P2X7R deletion did not affect Nrf2, xCT and GCLC levels under physiological conditions. Unexpectedly, LPS increased Nrf2 degradation and xCT expression, which were abrogated by P2X7R deletion. Furthermore, LPS did not affect GCLC levels in both *P2X7R^+/+^* and *P2X7R^−/−^* mice. Considering the xCT-mediated cystine-glutamate shuttle [58], it is likely that xCT upregulation with unaltered GCLC expression may be an Nrf2 independent compensatory response to GSH depletion induced by LPS. Indeed, *Nrf2^−/−^* mice show no genotypic difference in xCT level [59]. γ-Tocopheryl quinone (a powerful chemotherapeutic agent as an oxidative metabolite of γ-tocopherol) can increase cellular GSH levels without any considerable change in GCLC but facilitates the availability of cystine through Nrf2-independent xCT induction [60]. xCT level in astrocytes is also upregulated by intracellular GSH depletion, independent of Nrf2 [61]. However, xCT constitutes a cystine-cysteine shuttle whereby cystine uptake drives cysteine release, and extracellular cysteine provided by this shuttle is necessary for the transfer of NO equivalents [62]. Therefore, our findings provide the possibility that LPS-induced xCT upregulation may participate in the clearance of SNO-proteins against P2X7R-mediated nitrosative stress but may exacerbate GSH depletion by excessive cysteine efflux. Further studies are needed to elucidate the role of xCT upregulation under neuroinflammatory conditions.

Glutamate and glutamine are equally required for GSH biosynthesis through the glutamate-glutamine cycle that is regulated by GLS and GS [32]. LPS activates GLS accompanied by increased NO production [40,63]. The present data also demonstrate that LPS increased GLS protein level in *P2X7R^+/+^*, but not *P2X7R^−/−^* mice, although P2X7R deletion did not affect GLS expression under physiological conditions. Since xCT exchanges glutamate for cystine influx [14,15], GLS upregulation may increase glutamate gradients to facilitate xCT-mediated cystine uptake for GSH synthesis. Indeed, GLS hyperactivation increases glutamate release and xCT upregulation amplifies glutamate efflux [64,65]. In contrast, LPS reduces GS expression, which enhances the release of inflammatory mediators and leads to perturbation of the redox balance [66,67]. Consistent with our previous study [18], the present study shows that P2X7R deletion increased GS expression, but reduced SNO-GS levels under physiological conditions. LPS decreased GS expression, accompanied by the increased SNO-GS level in *P2X7R^+/+^* mice, while it did not affect them in *P2X7R^−/−^* mice. Since *S*-nitrosylation of GS leads to its degradation by the 20S proteasome [68], our findings indicate that P2X7R may decrease GS level by accelerating *S*-nitrosylation-mediated GS degradation under physiological- and post-LPS conditions. Considering that GS converts glutamate to glutamine [38], this GS downregulation may contribute to an increase in intracellular glutamate concentration representing an aberrant glutamate-glutamine cycle under inflammatory conditions. Interestingly, glutamine enhances Nrf2 and GSHS activities [25]. Therefore, it is likely that GS upregulation in *P2X7R^−/−^* mice may also play an important role in the preservation of Nrf2 and GSHS levels following LPS treatment. Taken together, our findings suggest that P2X7R may modulate LPS-induce neuroinflammation by regulating the glutamate-glutamine cycle.

ASCT2 is a glutamine:cysteine exchanger that participates in GSH biosynthesis [41,42]. *S*-nitrosylation inhibits ASCT2 activity [47] and leads to ASCT2 upregulation as an adaptive response [48]. In the present study, P2X7R deletion increased ASCT2 expression, but reduced SNO-ASCT2 level under physiological conditions. LPS decreased ASCT2 expression, accompanied by the increased SNO-ASCT2 level in *P2X7R^+/+^* mice, while it enhanced only SNO-ASCT2 level in *P2X7R^−/−^* mice. The present data also reveal that GS and SNO-GS levels had direct proportional relationships to ASCT2 and SNO-ASCT2 levels, respectively, in both *P2X7R^+/+^* and *P2X7R^−/−^* mice, indicating that GS activity/expression may regulate ASCT2 expression independent of P2X7R. Compatible with the reduced GS expression; therefore, our findings suggest that P2X7R may inhibit ASCT2-mediated glutamine:cysteine exchange under neuroinflammatory conditions, which would reduce GSH biosynthesis.

In the present study, P2X7R deletion relieved, not completely inhibited, the upregulations of iNOS expression and SNO-cysteine level induced by LPS. However, SNO-GS level was unaffected by LPS in *P2X7R^−/−^* mice. Although we cannot provide the underlying mechanisms of this phenomenon, the possibility would considerable. In contrast to the case of SNO-GS level, the present data show the increased SNO-ASCT2 level induced by LPS in both *P2X7R^+/+^* and *P2X7R^−/−^* mice. Therefore, it is likely that NO generated from iNOS may lead to *S*-nitrosylation of ASCT2 rather than GS due to the distinct affinity of NO bindings between ASCT2 and GS. The affinity test for NO binding to target proteins would be useful to understand the underlying mechanisms of nitrosative stress under neuroinflammatory conditions.

## 5. Conclusions

In the present study, we demonstrate that P2X7R deletion (1) attenuated iNOS upregulation and Nrf2 degradation induced by LPS, (2) decreased SNO-cysteine production under physiological and post-LPS treated conditions, (3) ameliorated the LPS-induced decreases in GSH, GSHS, GS and ASCT2 levels without altering GCLC level, (4) inhibited LPS-induced xCT and GLS upregulation, and (5) reduced *S*-nitrosylations of GS and ASCT2. These findings indicate that P2X7R may augment LPS-induced neuroinflammation by inducing Nrf2 degradation, aberrant glutamate-glutamine cycle and impaired cystine/cysteine uptake, which would inhibit GSH biosynthesis. Therefore, we suggest the targeting of P2X7R, which would exert nitrosative stress with iNOS in a positive feedback manner, and may be one of the important therapeutic strategies of nitrosative stress under pathophysiological conditions.

## Figures and Tables

**Table 1:** Primary antibodies and lectin used in the present study.

Antigen	Host	Manufacturer (Catalog Number)	Dilution Used
ASCT2	Rabbit	Alomone labs (Jerusalem, Israel) (#ANT-082)	1:500 (WB)
GCLC	Rabbit	Abcam (Waltham, MA, USA) (#ab190685)	1:2000 (WB)
GFAP	Mouse	Millipore ( Burlington, MS, USA) (#MAB3402)	1:2000 (IH)
GLS	Rabbit	Abcam (#ab93434)	1:1000 (WB)
GS	Mouse	Millipore (#MAB302)	1:1000 (WB)
GSHS	Rabbit	Abcam (#ab133592)	1:2000 (WB)
IB4		Vector (Los Altos, CA, USA) (#B-1205)	1:200 (histochemistry)
SNO-cysteine	Rabbit	Abcam (#ab94930)	1:1000 (IH)
iNOS	Rabbit	Novus Biologicals (Centennial, CO, USA) (#NB300-605)	1:100 (IH) 1:500 (WB)
Iba-1	Rabbit	Biocare Medical (Pacheco, CA, USA) (#CP 290)	1:500 (IH)
Nrf2	Rabbit	Abcam (#ab137550)	1:200 (IH) 1:1000 (IH)
xCT	Rabbit	Abcam (#ab175186)	1:1000 (WB)
β-actin	Mouse	Sigma (#A5316)	1:5000 (WB)

IH: Immunohistochemistry; WB: Western blot.

## Data Availability

The data presented in this study are available in the article and Supplementary Materials.

## Supplementary Materials

The following supporting information can be downloaded at: https://www.mdpi.com/article/10.3390/antiox11040778/s1, Figure S1: Full-length gel images of Western blot data in Figure 2A; Figure S2: Full-length gel images of Western blot data in Figure 4A; Figure S3: Full-length gel images of Western blot data in Figure 6B; Figure S4: Full-length gel images of Western blot data in Figure 7; Figure S5: Full-length gel images of Western blot data in Figure 8.
