# Supplementary material for: P2X7 Receptor Augments LPS-Induced Nitrosative Stress by Regulating Nrf2 and GSH Levels in the Mouse Hippocampus
Source: Antioxidants (Basel). 2022 Apr 13;11(4):778. doi: 10.3390/antiox11040778 (PMC9025791; doi:10.3390/antiox11040778)
Supplement: Supplementary file 1 [file antioxidants-11-00778-s001.zip › antioxidants-1671166-supplementary.pdf]

Supplementary Information

P2X7 Receptor Augments LPS-Induced Nitrosactive Stress by Regulating Nrf2 and GSH Levels in the Mouse Hippocampus

Duk-Shin Lee[1] and Ji-Eun Kim[1,*]

[1]Department of Anatomy and Neurobiology, Institute of Epilepsy Research, College of Medicine, Hallym University, Chuncheon 24252, South Korea

Running title: Role of P2X7R in LPS-induced nitrosactive stress

* Correspondence to: J.-E Kim, Department of Anatomy and Neurobiology, College of Medicine, Hallym University, Chunchon, Kangwon-Do 24252, South Korea; Tel: +82-33-248-2522; Fax: +82-33-248-2525;

E-mail: jieunkim@hallym.ac.kr

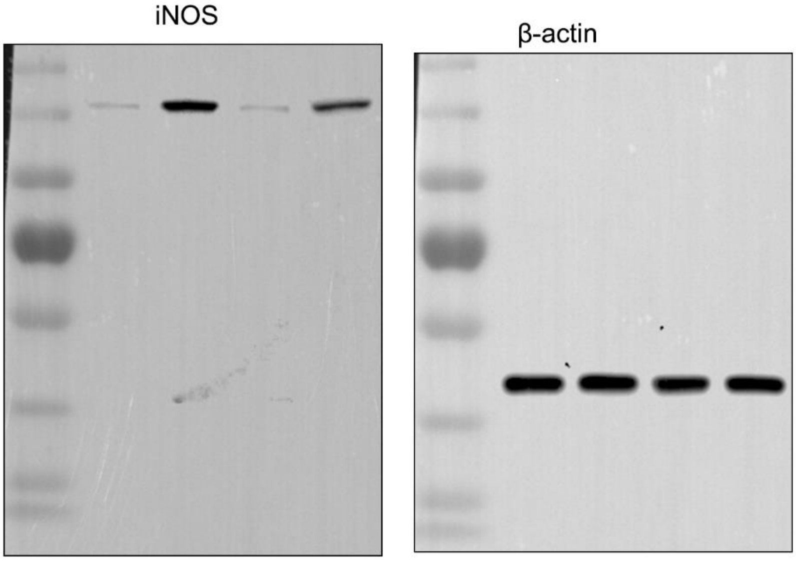

Supplementary Figure S1. Full-length gel images of Western blot data in Figure 2A.

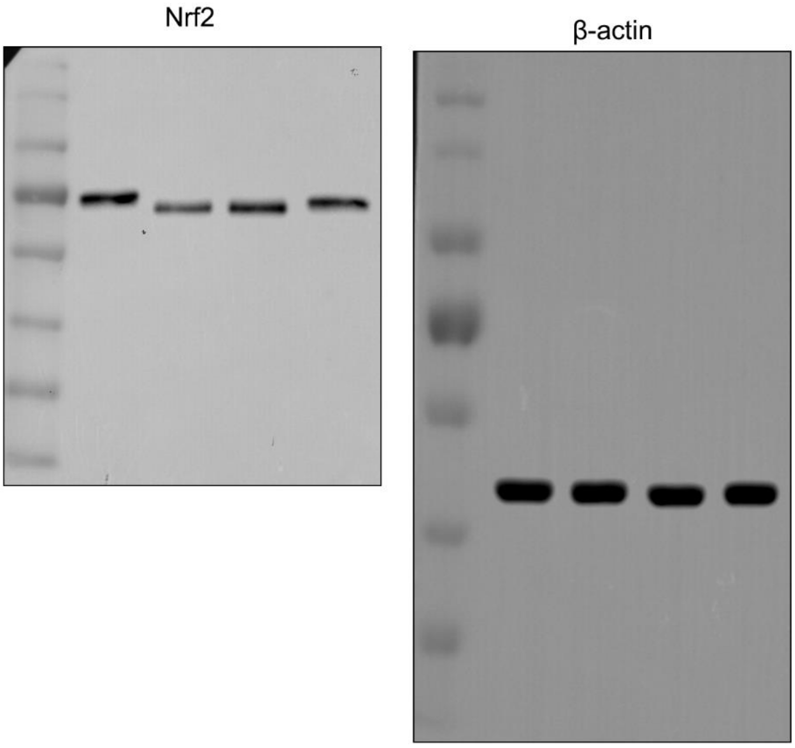

Supplementary Figure S2. Full-length gel images of Western blot data in Figure 4A.

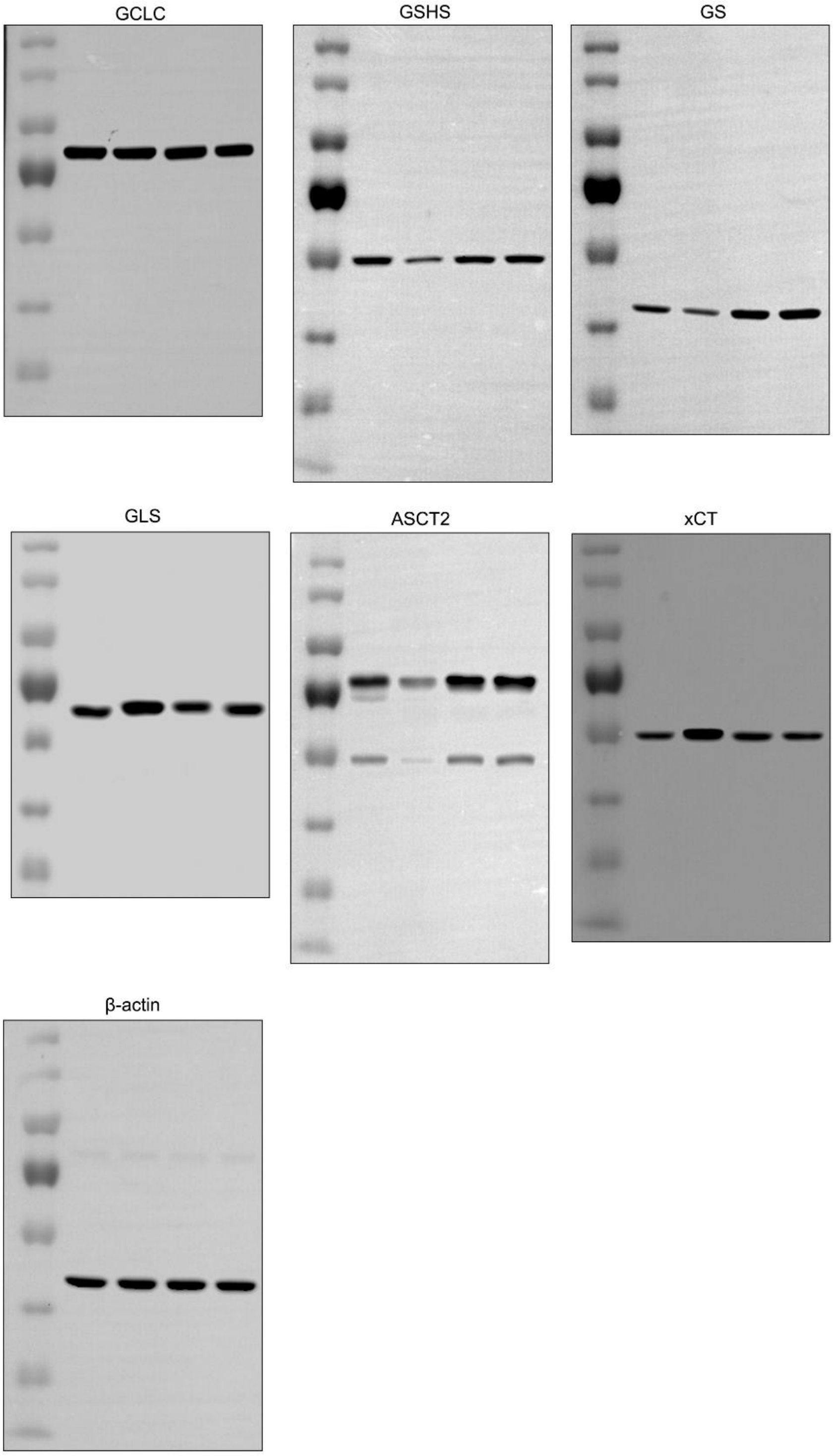

Supplementary Figure S3. Full-length gel images of Western blot data in Figure 6B.

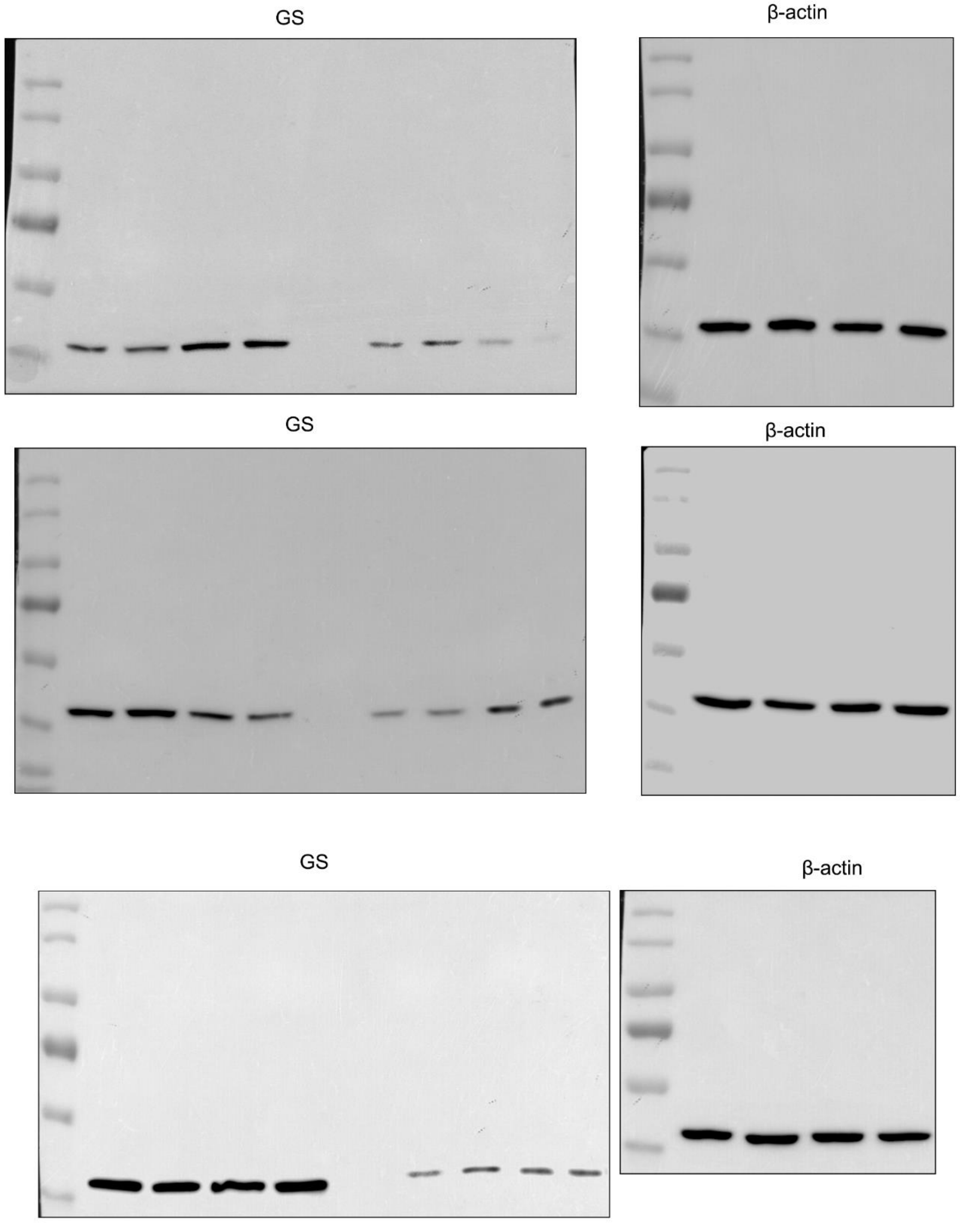

Supplementary Figure S4. Full-length gel images of Western blot data in Figure 7.

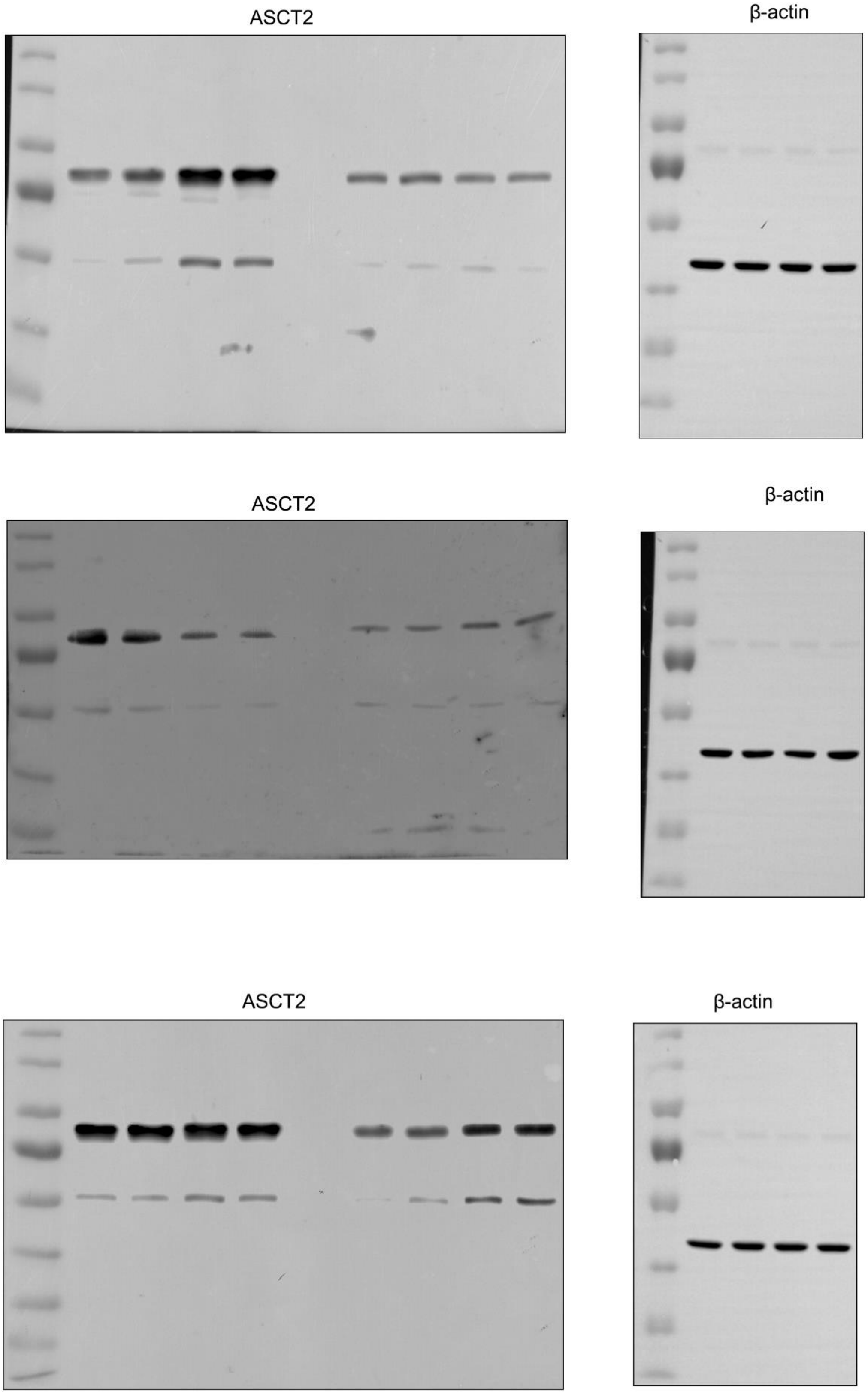

Supplementary Figure S5. Full-length gel images of Western blot data in Figure 8.
